# Supplementary material for: A Pilot Study: Favorable Effects of Clostridium butyricum on Intestinal Microbiota for Adjuvant Therapy of Lung Cancer
Source: Cancers (Basel). 2022 Jul 23;14(15):3599. doi: 10.3390/cancers14153599 (PMC9332558; doi:10.3390/cancers14153599)
Supplement: Supplementary file 1 [file cancers-14-03599-s001.zip › supplemental figures.pdf]

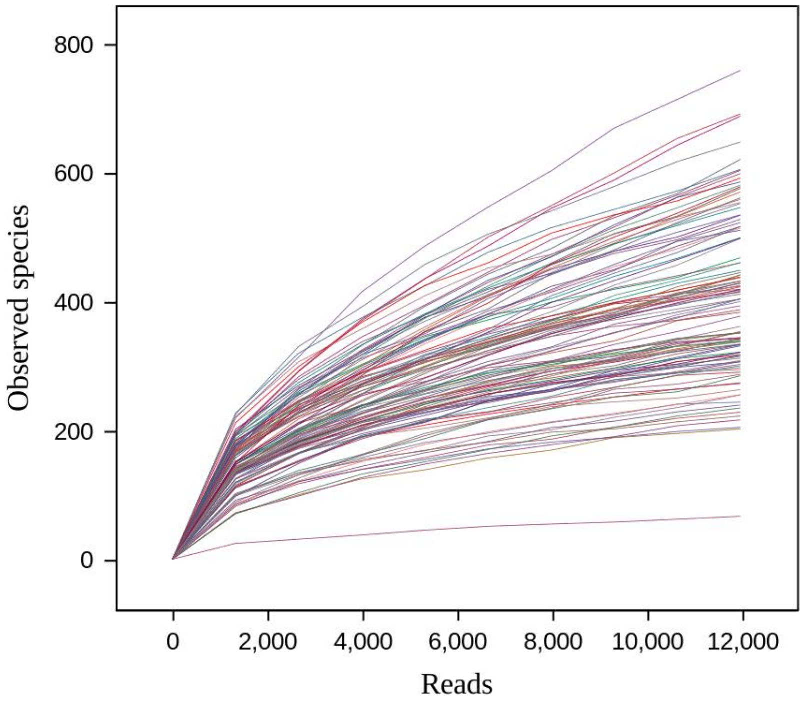

Figure S1. Rarefaction curve for NSCLC patients was generated at 97% similarity level.

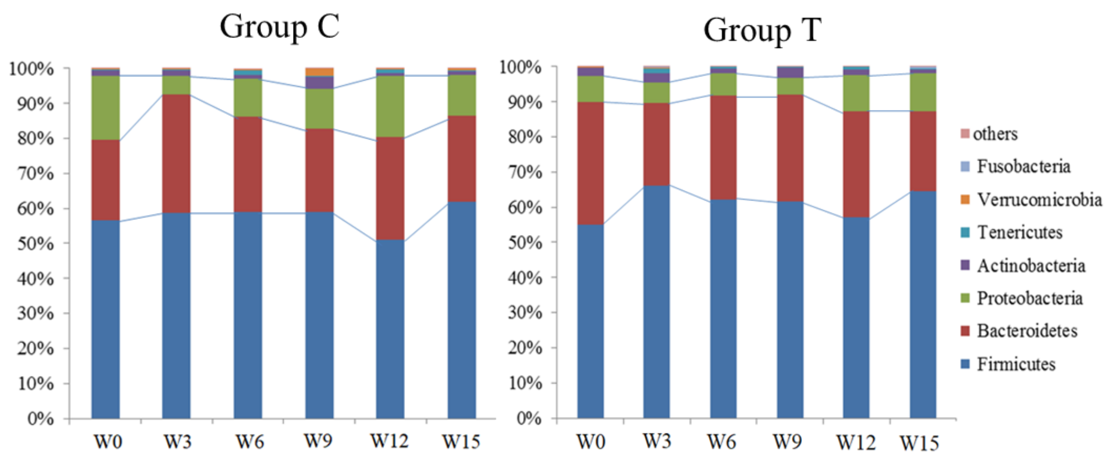

Figure S2. Microbial composition of C and T at the phylum level. The seven most abundant phyla of each group are shown.

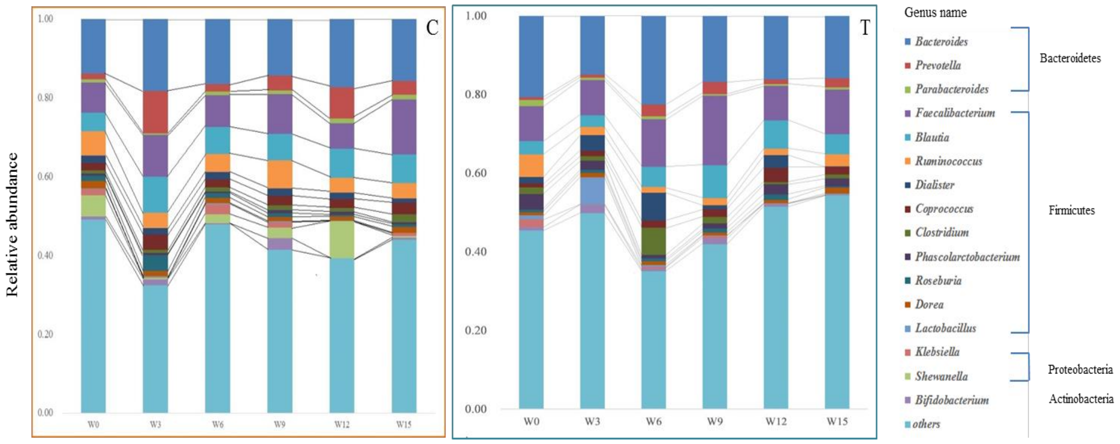

Figure S3. Changes in intestinal microbial composition at the taxonomical level between C and T during the treatment. C: placebo supplement; T: *Clostridium butyricum* supplement.

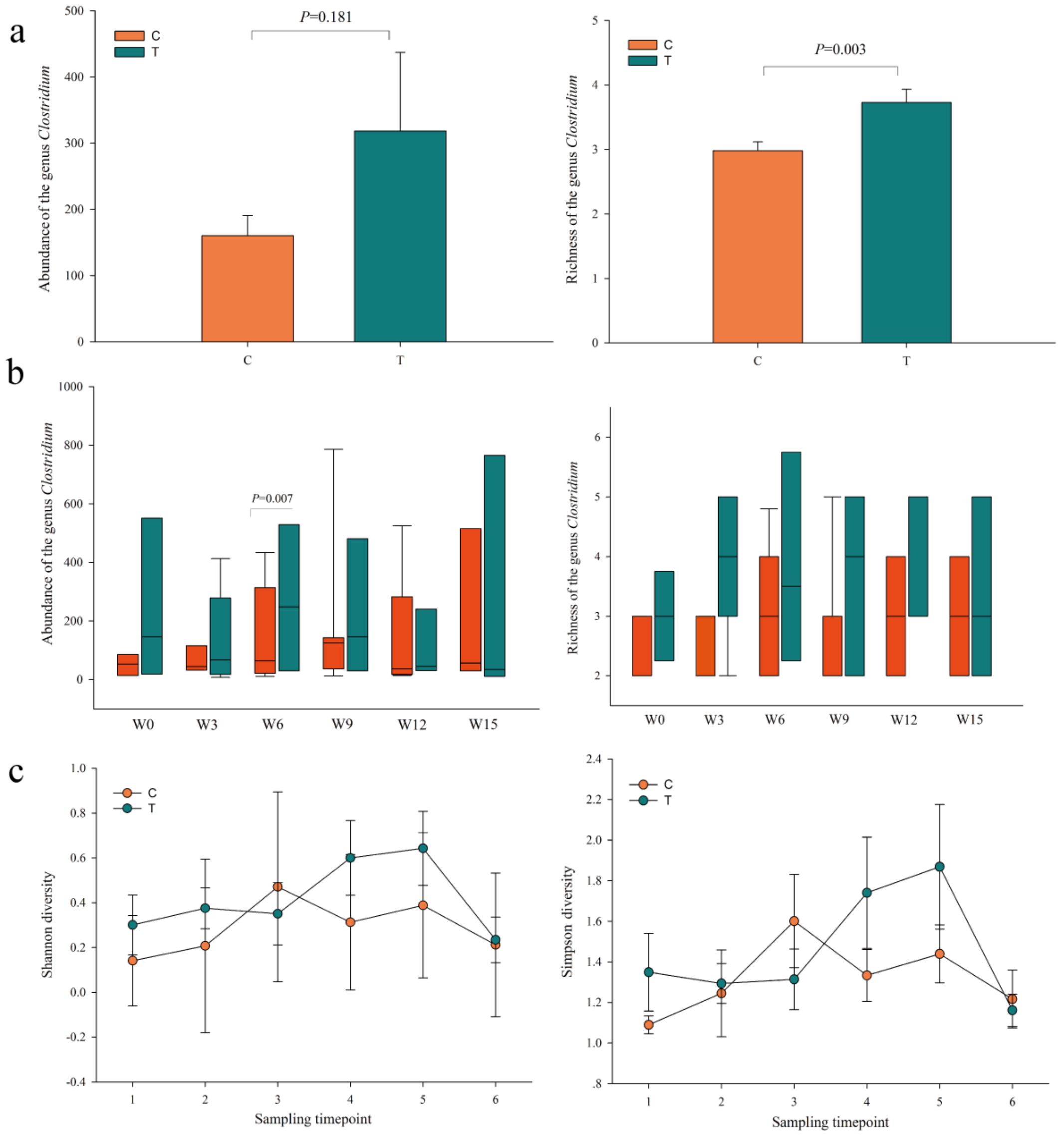

Figure S4. Differences in the genus *Clostridium* between C and T. (**A**) The total abundance (left) and richness (right). (**B**) The group abundance (left) and richness (right). (**C**) Shannon and Simpson diversity based on the genus *Clostridium*.

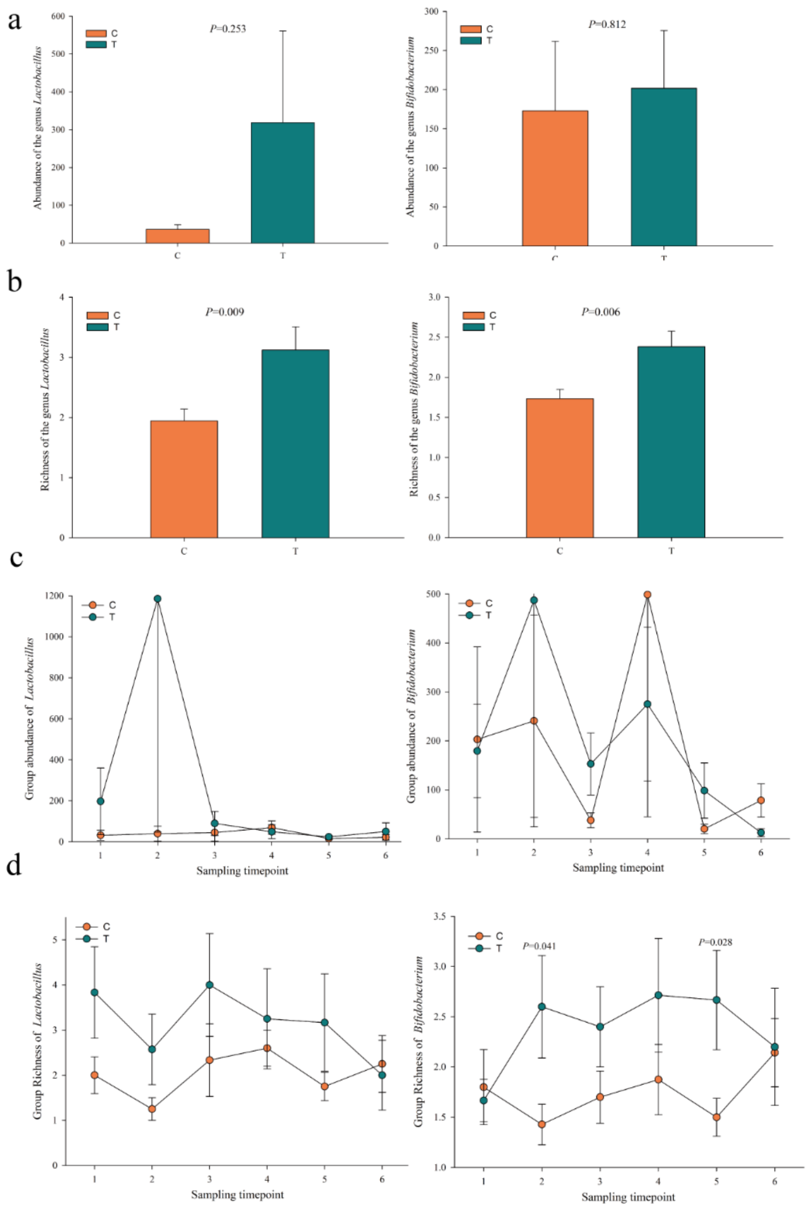

Figure S5. Differences in the genus *Bifidobacterium, and Lactobacillus* between C and T. (**A**) The total abundance; (**B**) The total richness; (**C**) The group abundance; (**D**) The group richness.

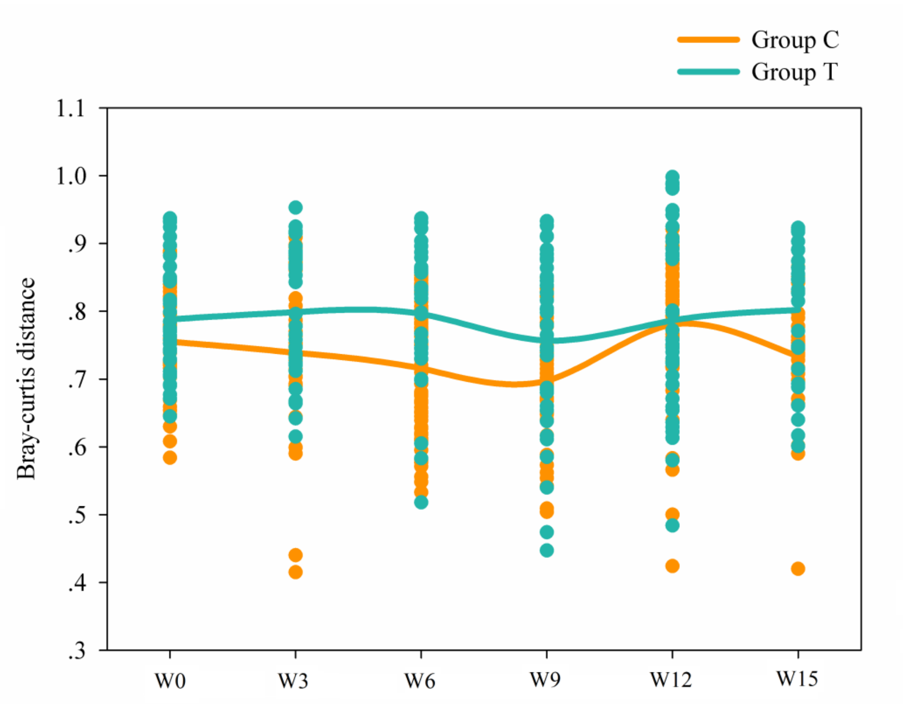

Figure S6. The Bray-Curtis distance calculated at the taxonomic level across individual microbiota of the same time point.

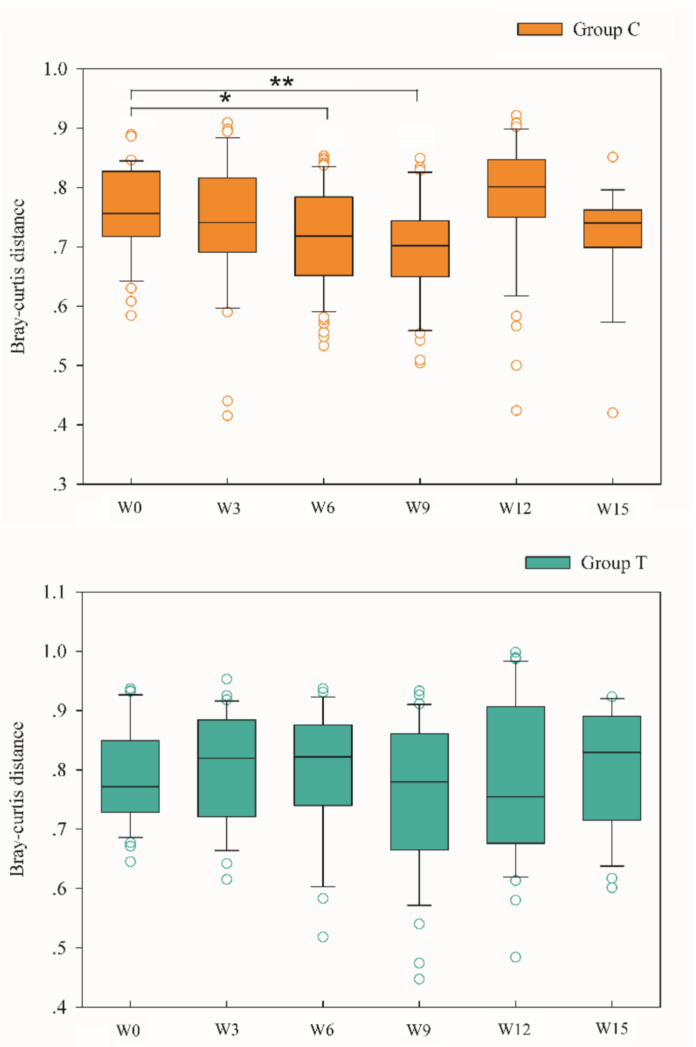

Figure S7. The Bray-Curtis distance between microbiota of each time point. Significance was measured using Wilcoxon rank-sum test (0.01 > P > 0.001, **; 0.05 > P > 0.01, *).

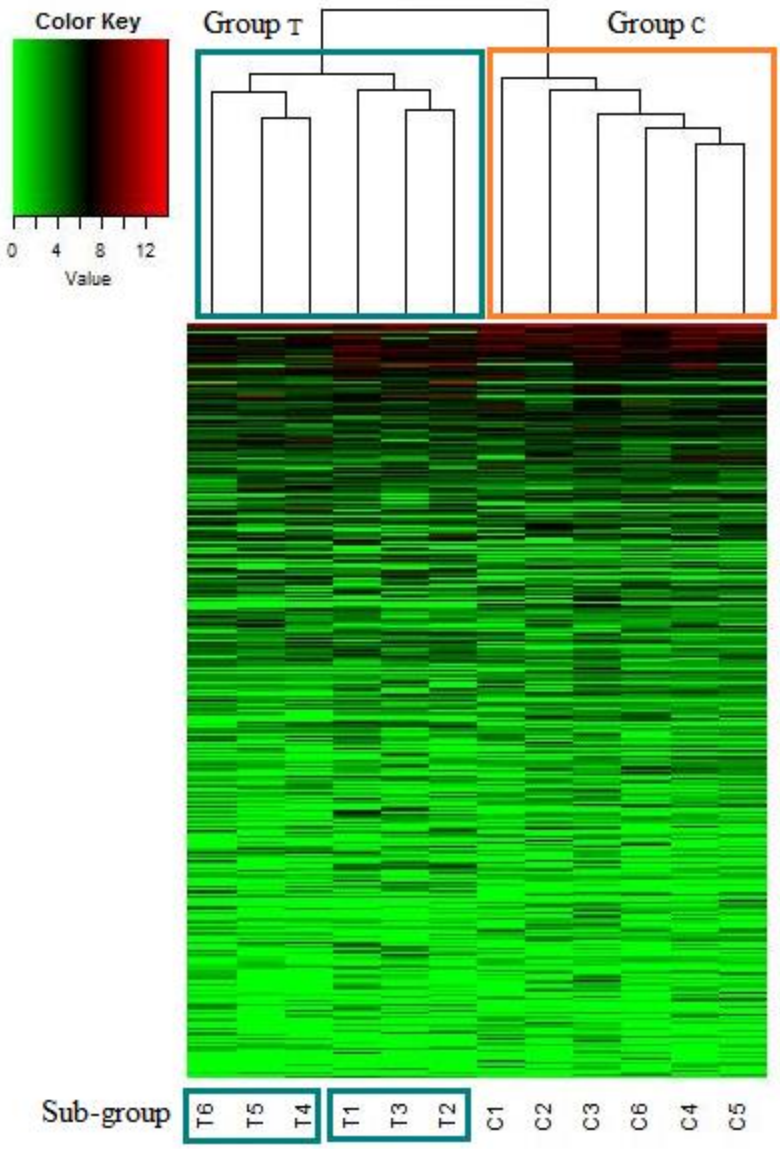

Figure S8. Clustering of intestinal microbiota in NS-NSCLC patients between C and T at the genus level. Note, T1-6 or C1-6 means that the treatment was carried out in the Week 0, 3, 6, 9, 12, 15.

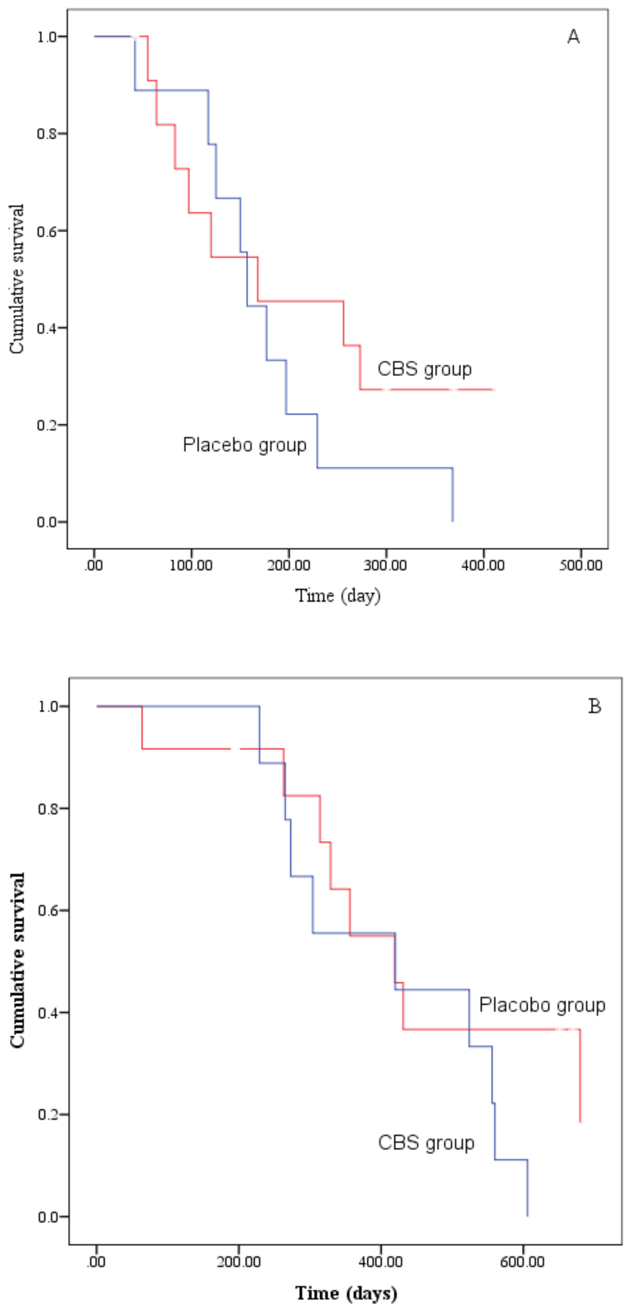

Figure S9. Kaplan-Meier survival curves for progression-free survival (**A**) and overall survival (**B**) patients between C and T. Censoring is indicated by the blank dot tic mark.
