# Supplementary material for: A Pilot Study: Favorable Effects of Clostridium butyricum on Intestinal Microbiota for Adjuvant Therapy of Lung Cancer
Source: Cancers (Basel). 2022 Jul 23;14(15):3599. doi: 10.3390/cancers14153599 (PMC9332558; doi:10.3390/cancers14153599)
Supplement: Supplementary file 1 [file cancers-14-03599-s001.zip › supplemental tables.pdf]

Table S1. Detailed clinical parameters of our study individuals.

Group	No.	Gender	Age	BMI	Smoking	BMI	EGFR (1=positive, 0=negative)	ALK (1=positive, 0=ngetive, 2=unknown)	Stage	ECOG	Date of informed consents	PFS(day)	OS(day)
C	1	Male	73	22.9	Yes	22.9	0	0	IIIB	1	2017.12.06	273	329
	2	Male	65	22.5	Yes	22.5	0	0	IIIB	1	2017.12.15	55	680
	3	Male	75	22.5	Yes	22.5	0	1	IV	1	2017.12.18	369	685
	4	Male	60	18.3	Yes	18.3	0	0	IV	1	2017.12.28	300	670
	5	Female	65	26.1	No	26.1	0	2	IV	1	2018.1.9	256	419
	6	Male	62	25.8	Yes	25.8	0	0	IV	1	2018.1.18	97	431
	7	Male	58	19.4	Yes	19.4	0	2	IIIB	1	2018.1.18	413	651
	8	Male	71	21.8	Yes	21.8	0	0	IV	1	2018.1.22	83	263
	9	Male	60	24.0	Yes	24.0	0	0	IV	1	2018.1.23	120	356
	10	Male	62	21.8	Yes	21.8	0	0	IV	1	2018.2.11	64	64
	11	Female	56	24.8	No	24.8	0	2	IV	1	2018.5.10	168	314
	12	Male	65	21.6	Yes	21.6	0	0	IV	2	2018.5.11	42	195
T	1	Male	68	21.5	Yes	21.5	0	2	IV	2	2018.3.6	368	606
	2	Male	55	24.7	Yes	24.7	0	2	IV	1	2018.3.26	157	560
	3	Female	69	19.2	No	19.2	0	2	IIIB	1	2018.4.18	197	273
	4	Male	61	21.3	Yes	21.3	0	0	IV	1	2018.4.23	229	556
	5	Female	50	18.8	No	18.8	0	0	IV	1	2018.4.28	42	265
	6	Male	61	19.4	Yes	19.4	0	0	IV	2	2018.5.3	117	304
	7	Female	29	23.0	No	23.0	0	0	IV	1	2018.5.18	150	229
	8	Female	52	26.0	No	26.0	0	0	IV	1	2018.5.28	125	524
	9	Male	48	24.4	Yes	24.4	0	2	IV	1	2018.9.3	177	420

Table S2. Detailed adverse events of our study individuals.

**Type of advent events**	**Frequency of advent events**																				
	**C1**	**C2**	**C3**	**C4**	**C5**	**C6**	**C7**	**C8**	**C9**	**C10**	**C11**	**C12**	**T1**	**T2**	**T3**	**T4**	**T5**	**T6**	**T7**	**T8**	**T9**
Anemia	2			2		3		1		2		3						1			
Neutropenia	10	3	8	1		4		1	1				1	1					1	1	
Thrombocytopenia	3			2		6								1	1	1					
Leukopenia	10	2	4	1		3								1					1	1	
Lymphocytopenia	8																				
High blood bilirubin		2	2		1						2										
Stomatitis																		1			
Periodontal pain							1	3													
Gingival bleeding		4																			
Toothache	2						3	1								1					
Dental caries		2																			
Epistaxis					3	2	3	2													1
Digestive tract perforation										1											
Gastritis		5			1										1						
Stomachache		1																			
Gastroesophageal reflux								1													
Abdominal distension				1	1			5													
Diarrhea		4	2		1	1				3											
Abdominal pain				2		1		2													
Constipation							1														
Appendicitis						1															
Upper respiratory infection				1		1	1					1									
Expectoration											1										
Cough			2			1	1								1						
Pneumonia				1						1	2							1			
Lung abscess												1		1							
Dyspnea						1															
Hematuria	1		5																		
Proteinuria		5	4				2	1			1	1		1	2						

Urinary tract infection					2												1				
Dysuria										1						1					
Skin rash						1	1												1		
Maculopapule		1										1									
Cutaneous pigmentation					1																1
Nail Discolouration					1																
Anorexia	2			1			1	3		1		1									
Vomit						1						1									
Nausea							1	1				2									1
Belching								2													
Depression								1													
Asthenia													1								
Malaise				1	3	1															1
Peripheral neuropathy	2	1	1	1			1	1		1			2					1	3		1
Alopecia	1	1	2	1	1		1	1			1	1	1		1			1	1		2
Pain	1	5	3	1	2								3		2			1			
Cancer pain	1									1							1				
Hoarseness		1																			
Bilateral inguinal hernia												1									
Insomnia		2	1					1								2					
Upper limb vein thrombosis								1													
Prostatic hyperplasia								1													
High alkaline phosphatase										1											
Prolonged APTT (activated partial thromboplastin time)												1									
Arthralgia	1	3	2		1			3													1
Myelosuppression															1	1					
High ALT						1				1											1
High glutamyl transpeptidase										1											
Hypertension		12		2	2		7						1		2	5				1	1
Hyperglycemia				1	1																
Hyperuricemia		1			1										1	1			1		
Hypernatremia				1																	

Hypermagnesemia	1		1		4		1	1		1	1		1			1					
Hyperkalemia				1																	
Hypercalcemia			1	2			1														
Hyperbilirubinemia	1																				
Hypercholesteremia								1													
Hyponatremia				2		1			1	2	1	2						1			1
Hypomagnesemia		1																			
Hypochloraemia										1		1									
Hypophosphatemia										1											
Hypokalemia											1										
Hypocalcemia	1		2				1														
Hypoalbuminemia		1				2	3		1	1	1	1	1	1	2	1	2	2			
Proctagra							1														
Hepatic function damage																		1			
Fever						1				1								1			
Allergy					1																
Vaginitis					1																

Table S3. The reads and OTUs of intestinal microbiota in 110 samples.

Group C	OTUs	Reads	Group T	OTUs	Reads
C1_1	233	51996	T1_1	240	47676
C2_1	236	51171	T2_1	169	42348
C3_1	198	44632	T3_1	223	33920
C4_1	258	29489	T4_1	134	31804
C5_1	295	49464	T5_1	187	27778
C7_1	93	43221	T6_1	197	27868
C10_1	158	41165	T7_1	147	34233
C11_1	224	37032	T8_1	344	33107
C12_1	149	47430	T9_1	281	35763
C1_2	199	53064	T1_2	244	47001
C2_2	212	40389	T2_2	161	44322
C3_2	238	55400	T3_2	181	43656
C4_2	179	51152	T4_2	96	36455
C6_2	233	39758	T5_2	175	29321
C8_2	290	47545	T6_2	235	28258
C9_2	222	42539	T7_2	181	32951
C10_2	177	33351	T8_2	355	36921
C11_2	195	40085	T9_2	268	29046
C1_3	246	52210	T1_3	233	41882
C2_3	244	43031	T2_3	105	40265
C3_3	239	52030	T3_3	226	43372
C4_3	305	51465	T4_3	123	34769
C5_3	329	46209	T5_3	211	29380
C6_3	230	38315	T6_3	225	28759
C7_3	142	45490	T7_3	172	43288
C8_3	289	33218	T8_3	367	33373
C9_3	283	39608	T1_4	157	48665
C10_3	225	35881	T2_4	202	46014

C11_3	294	42989	T3_4	200	41945
C12_3	281	42228	T4_4	143	45176
C1_4	269	53370	T5_4	127	28280
C2_4	232	41774	T6_4	215	28612
C3_4	237	51934	T7_4	157	30776
C4_4	246	50792	T8_4	353	33001
C5_4	277	45850	T9_4	251	29701
C6_4	189	32297	T1_5	26	36942
C7_4	142	44002	T2_5	229	44444
C8_4	218	44748	T3_5	229	41648
C9_4	290	40915	T4_5	160	32822
C10_4	160	40368	T5_5	212	37216
C1_5	217	44802	T6_5	249	36160
C2_5	215	58126	T7_5	150	33480
C3_5	201	51141	T8_5	241	32607
C4_5	228	52191	T9_5	266	29773
C5_5	314	42402	T1_6	190	37946
C6_5	236	41708	T2_6	169	37026
C7_5	173	39403	T3_6	258	41375
C8_5	265	37734	T5_6	150	39754
C9_5	229	47035	T6_6	222	35757
C10_5	270	37282	T7_6	188	29445
C1_6	241	54901	T8_6	325	30389
C2_6	218	43646	T9_6	249	30848
C3_6	230	46765			
C4_6	323	53941			
C5_6	282	43235			
C6_6	220	42013			
C8_6	224	45787			
C9_6	237	40925			

Table S4. Differences of the top seven phyla for NS-NSCLC between C and T.

Group	Firmicutes	Bacteroidetes	Proteobacteria	Actinobacteria	Tenericutes	Verrucomicrobia	Fusobacteria
C1-T1	0.86	0.16	0.14	0.58	0.43	0.50	0.84
C2-T2	0.14	0.06	0.76	0.68	0.41	0.34	0.52
C3-T3	0.60	0.70	0.21	0.74	0.43	0.33	0.40
C4-T4	0.73	0.42	0.08	0.88	0.36	0.25	0.35
C5-T5	0.39	0.92	0.37	0.17	0.57	**0.05**	0.87
C6-T6	0.74	0.78	0.87	0.86	0.98	0.12	0.36
C1-C2	0.80	0.06	0.10	0.74	0.87	0.55	0.39
C1-C3	0.72	0.47	0.25	0.73	0.27	0.71	0.35
C1-C4	0.76	0.85	0.33	0.53	0.66	0.31	0.36
C1-C5	0.43	0.38	0.92	0.42	0.28	0.59	0.29
C1-C6	0.50	0.84	0.42	0.75	0.79	0.58	0.29
T1-T2	0.13	0.18	0.67	0.83	0.36	0.35	0.27
T1-T3	0.37	0.54	0.73	0.51	0.40	0.46	0.76
T1-T4	0.47	0.65	0.38	0.73	0.53	0.34	0.84
T1-T5	0.81	0.64	0.60	0.76	0.27	0.32	0.31
T1-T6	0.22	0.14	0.37	0.38	0.33	0.34	0.35

Table S5. The differences of the top thirty genera for NS-NSCLC between C and T.

Genus	C1-C2	C1-C3	C1-C4	C1-C5	C1-C6	T1-T2	T1-T3	T1-T4	T1-T5	T1-T6	C1-T1	C2-T2	C3-T3	C4-T4	C5-T5	C6-T6
Bacteroides	0.37	0.50	0.92	0.60	0.75	0.40	0.81	0.61	0.31	0.45	0.30	0.52	0.32	0.55	0.58	0.92
Faecalibacterium	0.38	0.87	0.25	0.62	0.19	0.95	0.44	0.11	0.81	0.54	0.70	0.66	0.35	0.15	0.57	0.59
Blautia	0.24	0.46	0.49	0.44	0.24	0.77	0.52	0.18	0.09	0.31	0.47	0.10	0.59	0.68	0.59	0.42
Prevotella	0.14	0.86	0.35	0.20	0.54	0.81	0.11	0.25	0.20	0.46	0.38	0.11	0.42	0.81	0.40	0.70
Ruminococcus	0.44	0.53	0.80	0.37	0.44	0.23	0.17	0.19	0.17	0.38	0.88	0.20	**0.04**	**0.01**	**0.03**	0.37
Coprococcus	0.13	0.80	0.48	0.76	0.32	0.72	0.32	0.22	0.13	0.27	0.37	**0.05**	0.72	0.48	0.49	0.41
Dialister	0.81	0.97	1.00	0.90	0.63	0.40	0.33	0.75	0.36	0.41	0.86	0.34	0.38	0.41	0.32	0.23
Clostridium	0.89	0.80	0.72	0.83	0.31	0.55	0.31	0.96	0.83	0.49	0.40	0.52	0.30	0.53	0.60	0.39
Shewanella	0.27	0.45	0.57	0.59	0.31	0.20	0.81	0.20	0.20	0.20	0.25	0.13	0.08	0.20	0.14	0.24
Phascolarctobacterium	0.80	0.94	0.53	0.70	0.75	0.53	0.19	0.27	0.50	0.59	0.14	0.22	0.29	0.62	0.48	0.08
Roseburia	0.17	0.88	0.57	0.06	0.11	0.82	0.86	0.62	0.11	0.98	0.27	0.11	0.40	0.93	**0.05**	0.84
Dorea	0.69	0.62	0.50	0.49	0.77	0.38	0.60	0.97	0.63	**0.05**	0.38	0.82	0.58	0.29	0.65	0.85
Lactobacillus	0.55	0.75	0.38	0.91	0.83	0.39	0.55	0.34	0.31	0.42	0.30	0.35	0.42	0.62	0.68	0.47
Bifidobacterium	0.71	0.47	0.48	0.39	0.71	0.57	0.78	0.52	0.47	0.16	0.96	0.79	0.23	0.65	0.31	0.12
Veillonella	0.34	0.60	0.37	0.94	0.89	0.80	0.54	0.34	0.20	0.19	0.93	0.19	0.98	0.26	0.20	0.20
Parabacteroides	0.44	0.67	0.87	0.59	0.64	0.11	0.18	0.06	0.07	0.10	0.22	0.79	0.53	0.21	0.23	0.42
Klebsiella	0.29	0.75	0.84	0.28	0.56	0.16	0.27	0.26	0.19	0.16	0.88	0.55	0.49	0.58	0.27	0.39
Streptococcus	0.37	0.41	0.51	0.41	0.43	0.52	0.65	0.79	0.33	0.11	0.75	0.31	0.28	0.38	0.27	**0.05**
Ochrobactrum	0.63	0.80	0.55	0.74	0.38	0.35	0.58	0.22	0.40	0.99	0.93	0.57	0.30	0.19	0.26	0.42
Oscillospira	0.35	0.15	0.44	0.26	0.55	0.50	0.33	0.33	0.18	0.82	0.25	0.52	0.20	0.49	0.22	0.99
Lachnospira	1.00	0.92	0.55	0.73	0.72	0.41	0.19	0.88	0.77	0.80	0.66	0.58	0.29	0.19	0.85	0.63
Acinetobacter	0.59	0.20	1.00	0.65	0.72	0.27	0.95	0.18	0.25	0.98	0.69	0.35	0.35	0.19	0.16	0.48
Sutterella	0.92	0.82	0.65	0.57	0.73	0.72	0.72	0.49	0.83	0.21	0.47	0.51	0.44	0.70	0.95	0.43
Haemophilus	0.35	0.37	0.34	0.33	0.50	0.64	0.31	0.07	0.32	0.16	0.32	0.39	0.69	0.22	0.33	0.24
Akkermansia	0.47	0.71	0.31	0.59	0.58	0.35	0.46	0.34	0.32	0.34	0.50	0.34	0.33	0.25	**0.05**	0.12
Anaerostipes	0.19	**0.03**	0.45	0.44	0.95	0.54	0.40	0.83	0.48	0.15	0.20	0.28	0.33	0.30	0.43	0.38
Butyricimonas	0.30	0.26	0.26	0.92	0.24	0.48	0.41	0.32	0.32	0.76	0.29	0.36	0.32	0.33	0.97	0.97
Collinsella	0.38	0.51	0.33	0.85	0.97	0.46	0.34	0.77	0.57	0.67	0.31	0.15	0.53	0.27	0.53	0.19

Fusobacterium	0.33	0.34	0.36	0.32	0.71	0.28	0.64	0.49	0.27	0.35	0.67	0.55	0.45	0.36	0.97	0.35
Desulfovibrio	0.80	0.51	0.85	0.81	0.55	0.47	0.83	0.94	0.29	0.26	0.92	0.58	0.34	0.98	0.35	0.54

Table S6. Differences of alpha diversity of intestinal microbiota for NS-NSCLC patients between C and T.

Group	Simpson (*P* value)	Shannon (*P* value)
C1-T1	0.763	0.772
C2-T2	0.629	0.777
C3-T3	0.071	0.116
C4-T4	0.059	0.339
C5-T5	0.655	0.126
C6-T6	0.814	0.307
C1-C2	0.960	0.606
C1-C3	0.075	**0.035**
C1-C4	0.153	0.360
C1-C5	0.413	0.179
C1-C6	0.852	0.084
T1-T2	0.837	0.678
T1-T3	0.670	0.586
T1-T4	0.514	0.438
T1-T5	0.603	0.510
T1-T6	0.807	0.871

Table S7. Statistical analysis of differences in intestinal microbiota in NS-NSCLC patients between C and T based on the bray-cutis.

Sample	MRPP (Delta, *P* value)	Anosim (R, *P* value)	Adonis (R^2, *P* value)
C1-T1	0.772, 0.483	0.022, 0.338	0.059, 0.441
C2-T2	0.769, 0.127	0.047, 0.185	0.071, 0.160
C3-T3	0.748, 0.281	0.106, 0.102	0.059, 0.254
C4-T4	0.726, 0.138	0.056, 0.153	0.068, 0.156
C5-T5	0.784, 0.448	0.023, 0.654	0.055, 0.481
C6-T6	0.768, 0.833	0.060, 0.847	0.054, 0.848
C1-C2	0.747, 0.179	0.065, 0.156	0.069, 0.197
C1-C3	0.733, 0.713	0.019, 0.357	0.046, 0.619
C1-C4	0.725, 0.926	0.037, 0.701	0.040, 0.912
C1-C5	0.769, 0.932	0.079, 0.904	0.036, 0.913
C1-C6	0.745, 0.807	0.079, 0.887	0.051, 0.839
T1-T2	0.766, 0.483	0.007, 0.381	0.059, 0.443
T1-T3	0.722, 0.104	**0.152, 0.024**	0.070, 0.055
T1-T4	0.744, 0.386	0.057, 0.145	0.060, 0.330
T1-T5	0.765, 0.382	0.058, 0.179	0.061, 0.330
T1-T6	0.737, 0.118	0.011, 0.407	0.079, 0.163

Table S8. Differences of beta diversity in intestinal microbiota for NS-NSCLC patients between C and T.

Group	Beta diversity	*P* value
C1-T1	0.76-**0.79**	0.086
C2-T2	0.74-**0.80**	**0.019**
C3-T3	0.72-**0.80**	**0.001**
C4-T4	0.70-**0.76**	**0.021**
C5-T5	0.78-**0.79**	0.834
C6-T6	0.73-**0.80**	**0.006**

Table S9. Information on the module hub OTUs and the connector OTUs in C and T.

Group	Topological roles	ID	Zi	Pi	Phylum	Genus
C1	Module hub	OTU33	2.65	0	Bacteroidetes	*Bacteroides*
C6	Connectors	OTU7	0.065	0.704	Bacteroidetes	*Bacteroides*
		OTU13	2.249	0.684	Firmicutes	*Unclassified_Ruminococcaceae*
		OTU43	1.913	0.683	Firmicutes	*Roseburia*
		OTU78	2.342	0.648	Firmicutes	*Blautia*
		OTU51	-0.674	0.656	Bacteroidetes	*Bacteroides*
		OTU65	-1.043	0.640	Firmicutes	*Coprococcus*
		OTU150	-0.674	0.667	Firmicutes	*Unclassified_Lachnospiraceae*
		OTU131	1.448	0.649	Firmicutes	*Unclassified_Ruminococcaceae*
		OTU123	-0.689	0.667	Firmicutes	*Unclassified_Ruminococcaceae*
		OTU142	0.069	0.704	Firmicutes	*Unclassified_Ruminococcaceae*
		**OTU261**	**-0.554**	**0.661**	**Firmicutes**	***Coprococcus***
		OTU1710	1.584	0.649	Bacteroidetes	*Odoribacter*
		OTU218	-1.355	0.640	Firmicutes	*Faecalibacterium*
		OTU823	-0.304	0.642	Firmicutes	*Coprococcus*
		OTU831	0.000	0.625	Actinobacteria	*Bifidobacterium*
T6		OTU83	1.945	0.667	Firmicutes	*Unclassified_Lachnospiraceae*
		**OTU261**	**-0.392**	**0.625**	**Bacteroidetes**	***Odoribacter***
